# Advantageous Reactivity of Unstable Metal Complexes: Potential Applications of Metal-Based Anticancer Drugs for Intratumoral Injections

**DOI:** 10.3390/pharmaceutics14040790

**Published:** 2022-04-04

**Authors:** Aviva Levina, Debbie C. Crans, Peter A. Lay

**Affiliations:** 1 School of Chemistry, The University of Sydney, Sydney, NSW 2006, Australia; 2 Department of Chemistry and the Cell and Molecular Biology Program, Colorado State University, Fort Collins, CO 80523, USA

**Keywords:** cancer, intratumoral injection, platinum, vanadium, iron, titanium, gallium, ruthenium, Schiff base, nanocarrier formulation

## Abstract

Injections of highly cytotoxic or immunomodulating drugs directly into the inoperable tumor is a procedure that is increasingly applied in the clinic and uses established Pt-based drugs. It is advantageous for less stable anticancer metal complexes that fail administration by the standard intravenous route. Such hydrophobic metal-containing complexes are rapidly taken up into cancer cells and cause cell death, while the release of their relatively non-toxic decomposition products into the blood has low systemic toxicity and, in some cases, may even be beneficial. This concept was recently proposed for V(V) complexes with hydrophobic organic ligands, but it can potentially be applied to other metal complexes, such as Ti(IV), Ga(III) and Ru(III) complexes, some of which were previously unsuccessful in human clinical trials when administered via intravenous injections. The potential beneficial effects include antidiabetic, neuroprotective and tissue-regenerating activities for V(V/IV); antimicrobial activities for Ga(III); and antimetastatic and potentially immunogenic activities for Ru(III). Utilizing organic ligands with limited stability under biological conditions, such as Schiff bases, further enhances the tuning of the reactivities of the metal complexes under the conditions of intratumoral injections. However, nanocarrier formulations are likely to be required for the delivery of unstable metal complexes into the tumor.

## 1. Introduction

The treatment of inoperable cancers, particularly those of the brain, head and neck, lung or pancreas, by direct injection of cytotoxic and/or immunomodulating drugs into the tumor is currently transitioning from experimental procedures to mainstream clinical practice [1,2,3,4,5]. Detailed clinical guidelines for intratumoral injections (ITI) have been outlined [6], and hundreds of clinical trials are either underway or have been completed [7]. The treatment of unresectable metastatic melanoma by ITI of an oncolytic virus (T-VEC) has been approved by the Food and Drug Administration (FDA) for human clinical use [8]. A related technique, convection enhanced delivery (CED), which is based on intracranial injections of chemotherapeutic drugs to overcome the blood-brain barrier, continues to be extensively trialed for the treatment of malignant gliomas [9,10,11]. Another related technique, pressurized intraperitoneal aerosolized chemotherapy (PIPAC), is under development for the treatment of metastatic cancers of the digestive system [12,13]. One of the main aims of these techniques is to maximize the concentrations of cytotoxic drugs within the tumor and to minimize their concentrations in the blood, which reduces the systemic toxicity of the treatment [1,2,3,4,5,9,10,11,12,13]. While ITI, CED and PIPAC treatments are generally regarded as palliative rather than curative, they can be applied in combination with systemic chemotherapy to reduce the spread of metastases and significantly prolong the life of cancer patients [14]. Classical Pt(II)-based anticancer drugs (cisplatin, carboplatin and oxaliplatin) [15] are increasingly used in ITI, CED and PIPAC formulations both in pre-clinical studies [14,16,17,18,19,20,21,22,23,24,25,26,27,28,29,30,31] and in human clinical trials as shown in Table 1 [7,32].

Extensive changes in the speciation of most metal-based drugs typically occur after their administration, due to the abundance of potential biomolecular ligands and reducing (or less commonly, oxidizing) agents in biological fluids [33,34,35,36,37,38,39,40,41,42]. One possible solution for this problem is the design of substitutionally inert (mostly organometallic) complexes where the metal ion acts either as a scaffold to build a three-dimensional organic structure for selective binding to protein targets [43,44,45] or as a catalytic center for intracellular redox reactions [46,47,48]. Another approach is to use kinetically inert Pt(IV) (see Section 7) or Co(III) prodrugs, which can be converted to their more labile Pt(II) or Co(II) counterparts in the reducing the environment of solid tumors [42,49,50,51]. This approach is often proposed for the targeted delivery of biologically active organic molecules that are bound to such metal ions [51,52,53]. However, their administration by intravenous injection can result in the reduction of the metal ion by Fe(II) in red blood cells with premature release of the active components [54,55,56].

**Figure 1:**
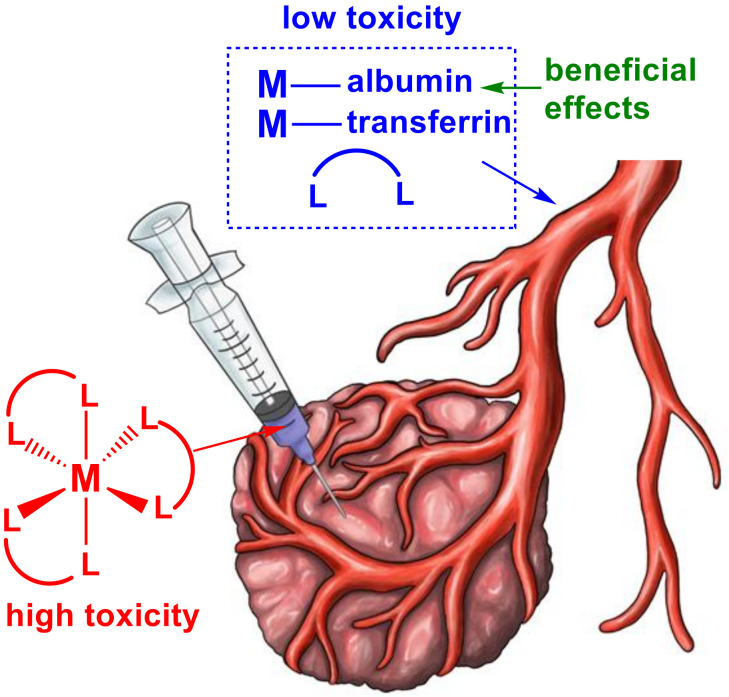
The principle of the use of reactive and unstable metal complexes in intratumoral injections [57]. Designations: M is the metal ion; and L are the ligands.

A novel concept that was recently proposed by our groups [57] involves the use of reactive metal complexes that have some stability but limited lifetimes in biological media. Such complexes are ideal agents for ITI and related delivery techniques of anticancer drugs. In this case, the binding of hydrophobic organic ligands to a toxic metal ion assists its efficient uptake into tumor cells and results in high cytotoxicity, while the decomposition products that are released into the blood stream consist of relatively non-toxic ligands and metal–protein complexes (Figure 1) [57]. This approach is expected to exhibit low systemic toxicity, similar to photodynamic therapy [58] or boron neutron capture therapy [59], where highly cytotoxic but short-lived agents are generated locally in the tumor tissue. Similar principles are also applied to organic anticancer prodrugs that hydrolyze in biological media with the formation of highly cytotoxic but short-lived active species [60,61]. Importantly, the decomposition products of some metal anticancer drugs are likely to have beneficial biological effects, as suggested previously for a V(V) complex with hydrophobic organic ligands [57]. In this review, we discuss a number of metal complexes with known anticancer properties that have potential for intratumoral applications.

## 2. Vanadium(V) Complexes

**Figure 2:**
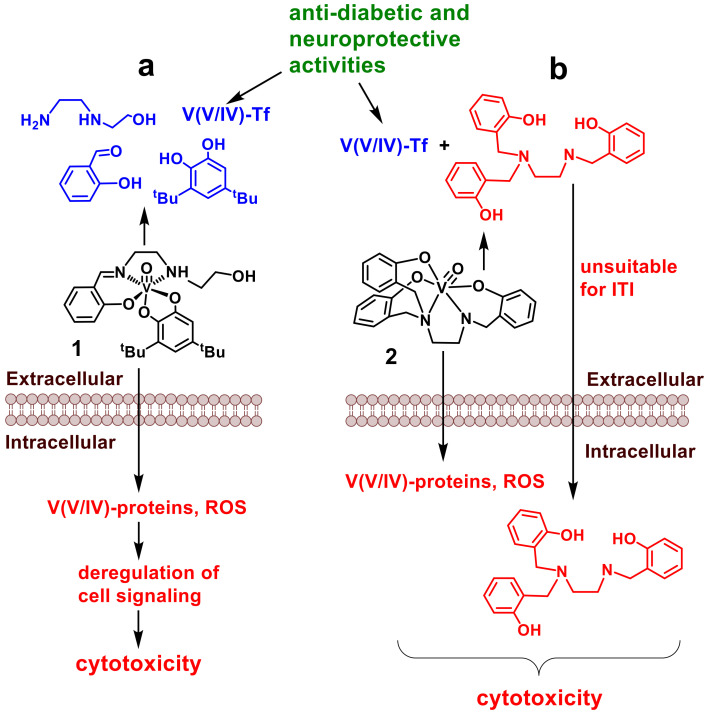
Proposed mechanisms of cytotoxic activity (red) and deactivation (blue) of V(V) complexes: (**a**) a complex with hydrolytically unstable Schiff base ligand (**1**) [57]; and (**b**) a complex with stable and cytotoxic salan-type ligand (**2**) [86]. Potential beneficial activities of the decomposition products are shown in green. Designations: Tf is apo-transferrin; ROS are reactive oxygen species and ^t^Bu is *tert*-butyl.

Anticancer activities have been reported for V(V/IV) complexes with many different structures [62,63]. The concept of using relatively unstable metal complexes for ITI, where the complexes had some stability and exerted high reactivity, was developed for a non-innocent oxidovanadium(V) complex with a tridentate Schiff base and a redox-active di-3,5-*tert*-butylcatecholato ligand (**1** in Figure 2a) [57,64]. Despite the vanadate–phosphate analogy [65,66], the nature of V−O bond in **1** and in other V(V/IV) complexes with organic ligands is closer to a triple than a double bond (2.5 < *n* ≤ 3, Figure 2a) due to the presence of one σ and two π bonds, and the bond is thus presented as a triple bond [67,68]. Due to the hydrophobic nature of the ligands [57,64] and sufficient stability of the coordination complex in biological media, **1** is efficiently taken up by cancer cell monolayers and causes high cytotoxicity (IC_50_ ~ 1–4 μM in 72 h treatments). Complex **1** is ~10-fold more toxic than cisplatin under the same conditions [57,64]. This effect is likely to be caused by changes in cell signaling that could originate from direct interactions of the cell membrane with V-complexes [69,70,71], inhibition of protein phosphatases by V-derivatives [66,72], as well as from V(V) reactions with cellular reductants that generate reactive oxygen species (ROS); see Figure 2a [73,74,75]. In parallel, rapid decomposition of **1** in cell culture medium occurs (half-life, ~30 s at 37 °C) [57], which involves hydrolysis of the Schiff base ligand, the release of oxidovanadium(V) species and their binding to serum proteins, predominantly transferrin (Tf, Figure 2a) [37,40,76,77]. This decomposition leads to a decrease in cytotoxicity by an order of magnitude, due to the low cellular uptake of V-Tf adducts and low cytotoxicity of the ligand fragments [37,57]. Furthermore, V-Tf adducts are likely to be involved in the beneficial biological activities of V, such as the well-known antidiabetic [78,79] and the recently demonstrated neuroprotective and neurostimulatory [80,81,82] effects. The latter activities, together with the favorable cytotoxicity ratio of fresh and decomposed **1** in human glioma multiforme (T98g) cells, led to the suggestion [57] that **1** can be used in the ITI formulations for this aggressive form of brain cancer. This suggestion is supported by the recently demonstrated low acute oral toxicity of **1** in mice [83]. Neuroprotective and neurostimulatory activities of the decomposition products of **1** may help to fight the neurological and cognitive disorders that commonly occur from cancer itself, or from standard chemotherapy [84,85].

For comparison, the parent analog of **1** without *tert*-butyl substituents in the catechol ligand (the simple catechol) decomposes completely within a few seconds in the cell culture medium and is not taken by the cells to a significant extent [64]. Further developments in this field will involve tuning the hydrophobicity and aqueous stability of mixed-ligand V(V) complexes. This will enable optimization of their cellular uptake and decomposition rates and cytotoxic activities for the use in ITI and related techniques [87].

Like **1**, V(V) complexes with reduced Schiff base (salan-type) [86,88] ligands, such as **2** in Figure 2b, are efficiently taken into cultured human cancer cells and are highly cytotoxic [86,89]. Unlike for **1**, the cytotoxicity of **2** is predominantly due to the release of hydrolytically stable ligands, extracellularly and/or intracellularly (Figure 2b) [86]. Similar ligand-based cytotoxicity mechanisms have been proposed for V(V/IV) complexes with typical hydrophobic and cytotoxic chelating ligands, such as 1,10-phenanthroline or 8-hydroxyquinoline [39,90,91]. The release of stable and highly cytotoxic ligands into the blood stream is likely to lead to significant systemic toxicity that complicates the use of **2** and other V(V) complexes with stable cytotoxic ligands in ITI (Figure 2b). However, salan-type ligands in V(V) complexes can also be relatively non-toxic [92], which emphasizes the need for comparative biological activity studies of metal complexes and the corresponding free ligands [36].

Schiff bases, particularly those derived from salicylaldehyde and diamines (salen-type ligands), have long been considered a staple of coordination chemistry. Numerous metal complexes of these ligands have undergone biological activity assays, but none seem to have entered advanced preclinical development, as of yet [88,93,94]. Although the hydrolysis of Schiff bases to the original aldehyde and amine components in neutral aqueous solutions has long been known [95,96], its implications for biological activities of metal Schiff base complexes have not been recognized until recently [88]. For instance, the formation of aldehyde and amine precursors of the Schiff base ligand during the dissolution and subsequent decomposition of **1** in water (Figure 2a) has been demonstrated by ^1^H NMR spectroscopy [64]. The reactivity of the complex and ligand cleavage and V(V) release (Figure 2a) is responsible for the short lifetime of **1** under biologically relevant conditions, which forms the basis of the proposed use of **1** in ITI [57].

## 3. Iron(III) Schiff Base Complexes

**Figure 3:**
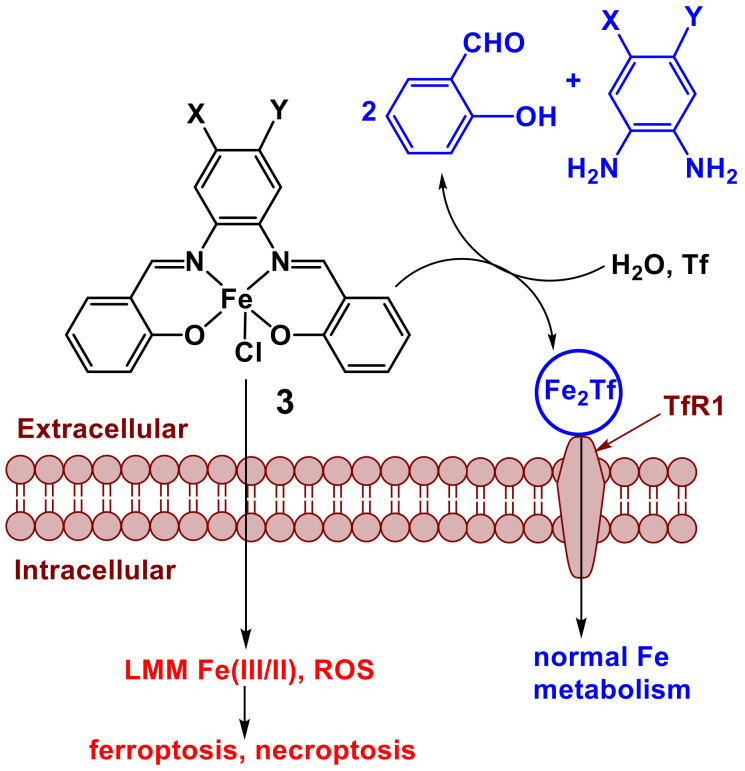
Proposed mechanism of cytotoxic activity (red) and deactivation (blue) of a Fe(III) complex with Schiff base ligand (**3**) [97,98]. Various substituents (X and Y) in the ligand were used, including halogens, CH_3_, OCH_3_, NO_2_, or C(O)XR, where X is O or NH, and R is Et, *n*-Pr or *n*-Bu [97,98]. The anticipated hydrolysis of the Schiff base ligand was not reported in the original articles and is based on the data reported for V(V/IV) Schiff base complexes [57,64]. Designations: Tf is apo-transferrin; TfR1 is transferrin receptor 1; LMM is low-molecular-mass; and ROS are reactive oxygen species.

Complexes of Fe(III) with salen-type ligands (**3** in Figure 3) [97,98,99,100] have recently been highlighted because of their ability to induce uncommon modes of cancer cell death, ferroptosis and necroptosis. Such modes of toxicity reduce the chance of the development of drug resistance [101]. These Fe(III) complexes are thought to bypass normal cellular Fe uptake and metabolism pathways by entering the cell through passive diffusion, which leads to the formation of highly reactive low-molecular mass (LMM) Fe(III/II) complexes and ROS (Figure 3) [97,98,99,100]. Although hydrolysis of the ligands has not been reported in the original articles, it is likely to contribute to the decomposition of **3** and related complexes in an extracellular medium. This would assist the binding of the released Fe(III) to Tf (Figure 3) [102], which has been observed experimentally [98]. Furthermore, the release of Fe from **3** and its binding to Tf and other biomolecules is likely to be assisted by the reduction of Fe(III) to Fe(II) in the hypoxic environment of solid tumors [1,50,55]. 

The resultant Fe(III)-loaded Tf can then enter cells via a canonical pathway through the binding to its cell surface receptor (TfR1 in Figure 3), followed by receptor-mediated endocytosis [102,103]. Apart from delivering essential Fe into the cells, Fe(III)-Tf binding in the blood plays a protective role by ensuring that no adventitious low-molecular-mass Fe species enter cells and cause excessive oxidative stress [37,102]. Therefore, the amount of Fe that enters cells through Tf-mediated uptake is expected to be lower than that delivered by the passive diffusion of a hydrophobic Fe(III) complex (Figure 3) [37,104].

The flexibility of salen-type ligands to diverse chemical modifications [88,93,94] offers possibilities for the design of Fe(III) complexes with suitable ratios of cellular uptake versus extracellular decomposition rates (Figure 3) for ITI. The use of an essential metal ion, such as Fe(III), enables the exploitation of the natural metal-binding capacity of extracellular fluids, including proteins (mainly transferrin and albumin) and low-molecular-mass ligands (such as citrate and phosphate) [36,40,102] to reduce the possibility of unwanted side effects. Schiff base ligand design can also be used to enable pH-dependent prodrug activation in the acidic extracellular environment surrounding solid tumors [105]. In addition to Schiff bases, other common transition metal ligands, such as (thio)semicarbazones, contain potentially hydrolysable imine functionalities [106]. These compounds are generally stable under physiological conditions and biologically active in their own right, or through the coordination to endogenous Fe(III) and Cu(II) [107,108]. Nevertheless, the possibility of metal- or enzyme-catalyzed hydrolysis of (thio)semicarbazone complexes [107,109] in biological media has potential use in ITI.

## 4. Titanium(IV) Complexes

**Figure 4:**
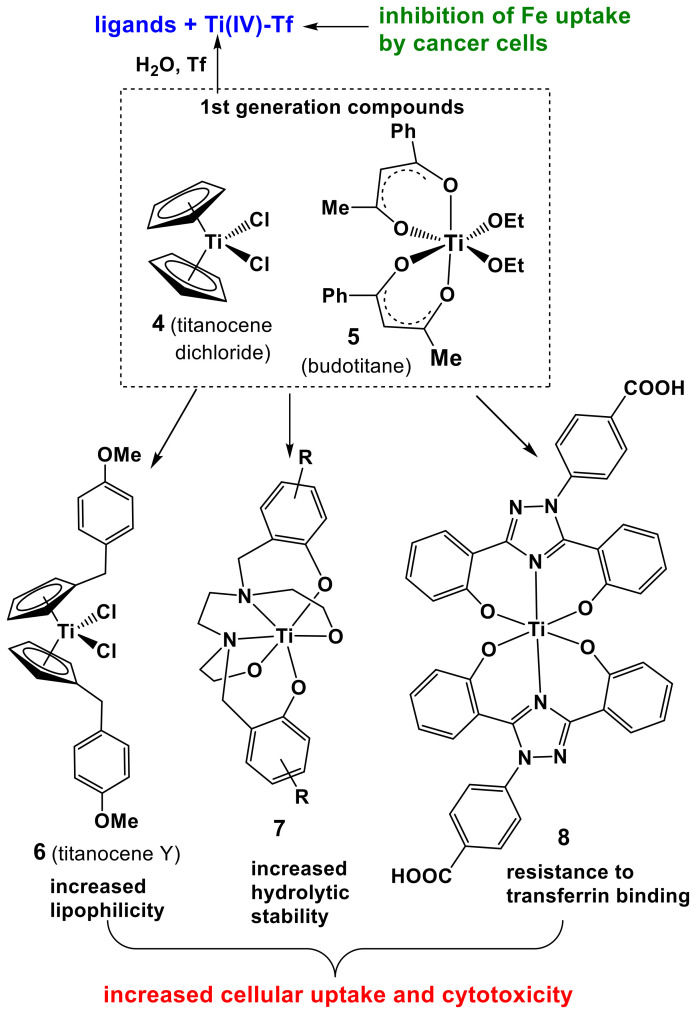
Typical first-generation (**4**, **5**) and second-generation (**6**–**8**) anticancer Ti(IV) complexes [112,116]. Likely decomposition products in an extracellular medium are shown in blue (Tf is apo-transferrin), and their potential beneficial activity is shown in green.

Titanocene dichloride and budotitane (**4** and **5** in Figure 4) were two of the earliest metal complexes after cisplatin to be developed as potential anticancer drugs in the late 1970s. The design was based initially on their structural similarity with cisplatin with two labile chlorido or ethanolato ligands in a *cis* arrangement [110,111,112]. Unfortunately, these complexes did not progress beyond phase I clinical trials because of formulation problems and dose-limiting nephrotoxicity [113]. Notably, **4** and **5** showed low systemic toxicity in animal studies, which is consistent with the generally low toxicity of Ti [112]. Nevertheless, the anticancer activities of **4** and **5** were attributed to the Ti(IV) ion, since this is the only structural element shared between the two complexes (Figure 4). A wide range of effects of Ti(IV) complexes was observed at the cellular level, including induction of apoptosis and paraptosis, inhibition of mitochondrial activity and inactivation of topoisomerases, but the origin of these effects remained uncertain [114]. Recently, interference with the Fe metabolism has emerged as the most likely underlying mechanism of Ti(IV) anticancer activity [115,116,117,118].

The complicated reactivity of Ti(IV) under biologically relevant conditions has been reviewed recently [117,119]. Complexes **4** and **5** are likely to decompose within seconds after intravenous injection with the formation of a mixture of low-molecular-mass hydrolysis products and Ti(IV)-protein adducts [112,117,119]. Extracellularly, Ti(IV) binds strongly and specifically to the Fe(III) binding sites of Tf [102,117]. This binding is mediated by citrate that helps to maintain Ti(IV) in a soluble form in neutral aqueous solutions [115]. Dependent on the nature of ligands, Ti(IV) complexes can also bind non-covalently to serum albumin [117,118]. Although Ti(IV)-Tf adducts can bind to cell surface TfR1 and enter cells through receptor-mediated endocytosis, similarly to Fe(III)-Tf (Figure 4), this uptake is less efficient compared with the passive diffusion of hydrophobic Ti(IV) complexes through the cell membrane [115]. Intracellularly, Ti(IV) complexes are likely to lose their ligands and to displace Fe(III) from the active sites of crucial enzymes, such as ribonucleotide reductase [104,116,117]. 

Many second- and third-generation anticancer Ti(IV) complexes were developed with the aim to slow down the rate of decomposition in the extracellular medium and to increase cellular uptake and cytotoxicity [112,118,120]. Typical examples (Figure 4) include increasing lipophilicity of cyclopentadiene ligands (**6**, titanocene Y) [121], using hexadentate ligands to easily exclude hydrolyzable groups (**7**) [122] and using ligands that mimic Tf binding sites to prevent extracellular Ti(IV) binding to Tf (**8**) [116]. It should be noted that the salan-type ligand in **7** is likely to be cytotoxic in its own right [86,88], which means that this complex is unlikely to be suitable for ITI. Some of the complexes shown in Figure 4, as well as other anticancer Ti(IV) complexes described in the literature [112], may be suitable for ITI if the ligand is sufficiently nontoxic. A possible additional advantage of the formation of Ti(IV)-Tf adducts during the decomposition of such complexes outside the cells (Figure 4) is the decrease in availability of Fe(III)-Tf to rapidly growing cancer cells since they have a high metabolic demand for Fe [102,123].

## 5. Gallium(III) Complexes

**Figure 5:**
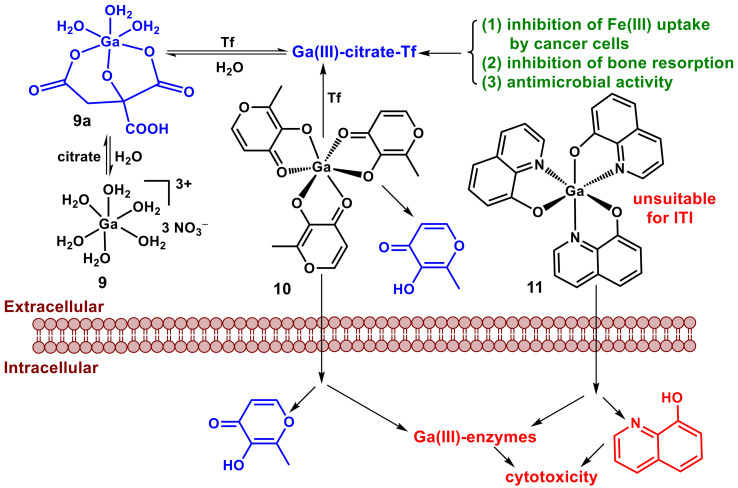
Proposed mechanisms of cytotoxic activity (red) and deactivation (blue) of Ga(III) complexes (**9**–**11**; Tf is apo-transferrin) [124,125,126]. The mono-citrato Ga(III) complex **9a** represents one of the many possible structures of Ga(III)-citrato complexes [128,129], and **10** and **11** are the *fac* isomers but other species may be present. Potential beneficial activities of the extracellular decomposition products are listed in green.

Unlike for Ti(IV) complexes, the use of Ga(III) complexes as anticancer drugs was originally based on the concept of chemical similarity of Ga(III) to high-spin Fe(III). This was expected to lead to the disruption of Fe metabolism in rapidly growing cancer cells [113,124,125,126]. Inorganic Ga(III) salts (nitrate or chloride, **9**, Figure 5), injected intravenously in citrate-buffered solutions [127] (shown schematically as **9a**, Figure 5) [128,129], reached phase II clinical trials for non-Hodgkin’s lymphoma and advanced melanoma [113]. The use of Ga(III) nitrate was later approved for the treatment of cancer-related calcium overload, but it is currently not used in the clinic [113]. Radiolabeled ^67^Ga(III)-citrate injections are still used in the diagnostics of cancer and inflammation, although they are increasingly replaced by ^18^F-based positron emission tomography (PET) scans [113,125]. Complexes with hydrophobic organic ligands, such as maltol or 8-hydroxyquinoline (**10** and **11**, respectively, in Figure 5) were designed to increase the bioavailability of Ga(III) for their potential use as oral anticancer drugs [113,125]. While clinical trials of **10** were discontinued after phase I/II, **11** is still in active trials and has shown promising results against renal cell carcinoma [113].

The cellular uptake of Ga(III) is generally thought to occur through Tf binding and interactions of the resultant Ga(III)-Tf adducts with TfR1 (similar to that for Fe(III) in Figure 3) [124], although the ability of Ga(III)-Tf to bind strongly to TfR1 has been disputed [130]. Speciation studies in bovine serum and in cell culture medium by X-ray absorption spectroscopy showed that **9** was bound to serum proteins, particularly albumin and transferrin, within minutes at 37 °C, **10** decomposed over several hours, and **11** reached partial decomposition after 24 h under these conditions [131,132,133]. These data suggest that **11** was more likely than **10** to enter cells intact through passive diffusion (Figure 5), although both complexes underwent extensive metabolic changes upon entering the cells [131,132,133]. The two complexes also differ in the biological activity of their ligands: maltol in **10** is considered non-toxic and is approved as a food additive [78], while 8-hydroxyquinoline in **11** is cytotoxic, probably due to the binding of extracellular Cu(II) and its delivery into cells (Cu ionophore) [134].

The moderate stability of **10** in biological media [132,133] and the non-toxic nature of its ligands make this Ga(III) complex a more suitable candidate for potential use in ITI, compared with **9** or **11**. The potential beneficial activities of the decomposition products of **10** (Figure 5) include decreased availability of Fe(III) to rapidly growing cancer cells due to the binding of Ga(III) to Fe(III)-binding sites of Tf [124], in the same way as proposed for Ti(IV) (Figure 4) [102,117]. In addition, the ability of Ga(III) to inhibit bone resorption and Ca(II) release has been reported [135], but the link between Ga(III) and Ca(II) remains much less explored than the similarities between Ga(III) and Fe(III) [124,125]. Recently, inorganic Ga(III) salts and Ga(III) complexes with organic ligands have emerged as potent antibacterial and antifungal agents with low toxicity to animals and humans [136,137,138,139,140,141,142,143]. Such beneficial antimicrobial activities are likely to be based on the differences in both Fe and Ca metabolism between microbial and mammalian cells [138,144]. This activity can potentially be used to help fight opportunistic infections that commonly occur as a result of cancer treatment by chemotherapy [145].

## 6. Ruthenium(III) Complexes

**Figure 6:**
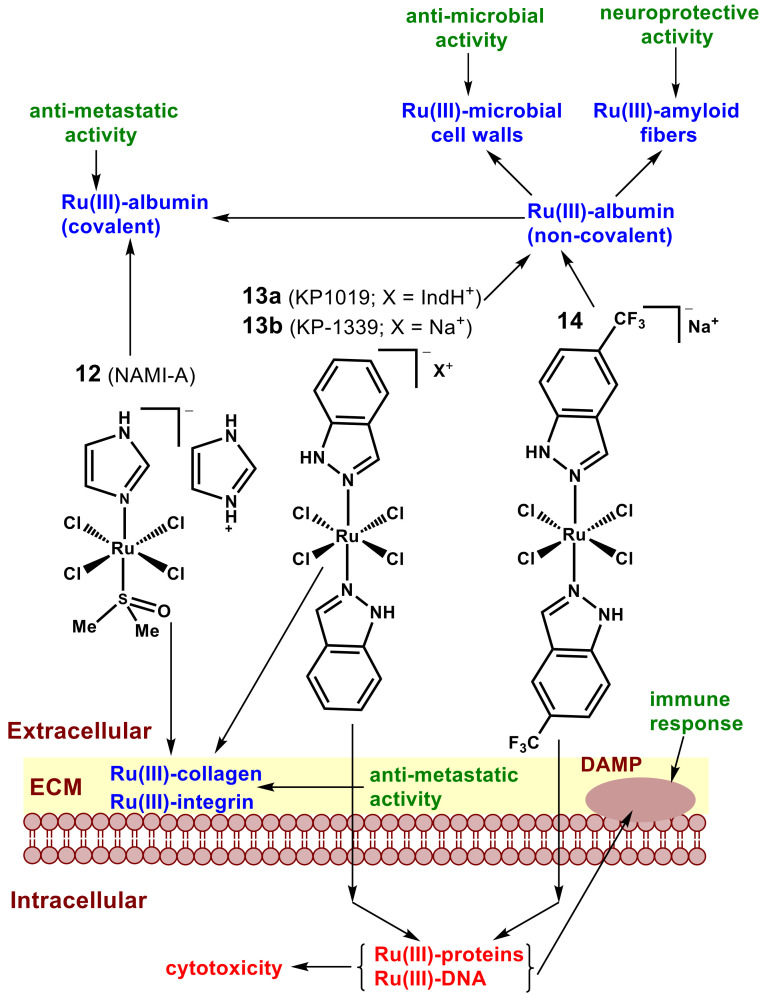
Structures of anticancer Ru(III) complexes that entered human clinical trials (**12**,**13**) and an investigational drug, **14** [113]. Their main modes of action in extra- and intracellular spaces (ECM is extracellular matrix, DAMP is damage-associated molecular pattern) are presented [33,113,147]. Intracellular cytotoxic species are shown in red, extracellular decomposition products are shown in blue, and their potential beneficial activities [151,154,155,156,165,166] are listed in green.

The anticancer activities of Ru(III) tetrachlorido complexes with axial *N*-heterocyclic ligands (Figure 6) have been extensively studied since the 1980s [33,113,146,147,148,149]. Two of the complexes, NAMI-A (**12**) and KP1019 (**13a**), reached human clinical trials but did not proceed beyond phase I/II. A more water-soluble analog of **13a**, KP1339 (**13b**, also known as NKP-1339, IT-139 and BOLD-100) is currently in phase I clinical trials in combination with established anticancer drugs [113,150,151,152,153]. The postulated mechanism of action of Ru(III) complexes involves the exchange of labile chlorido ligands for donor groups of various biomolecules, which leads to the binding to numerous intra- and extra-cellular targets [33,34,36,113,146,147,154,155]. Complexes with bulkier, more hydrophobic ligands, such as **13a**, rapidly enter the cells and cause significant cytotoxicity, while **12** binds predominantly to extracellular targets and is generally not cytotoxic (Figure 6) [33,113,146,147,156,157,158,159,160]. These complexes decompose in typical cell culture media or in blood serum within ~1 h (**12**) or ~4 h (**13a**) at 37 °C with the formation of predominantly Ru(III)-albumin adducts [156,160]. The binding of **12** to albumin involves the complete loss of the original ligands and the formation of covalent bonds with the side chains of the protein, and the resultant Ru(III)-albumin adducts are anti-invasive in cell culture assays [156,160]. The complete loss of the original ligands in NAMI-A during protein binding has been confirmed in several protein crystallography studies [161,162,163]. Fast non-covalent binding of **13a** to albumin occurs through hydrophobic interactions, followed by slower covalent binding [157,159,164]. The addition of trifluoromethyl groups to the indazole ligands in **14** enhances hydrophobic interactions with albumin, which results in increased stability in extracellular media and higher cellular uptake and cytotoxicity (Figure 6) [146].

Based on the results of animal experiments, a unique mode of action of **12** was proposed, in which the drug does not decrease the size of primary tumors but prevents the spread of metastases [147,148]. Covalent binding of **12** to cell surface integrins and to the components of extracellular matrix (ECM), such as collagens (Figure 6), can disrupt the cell–cell and cell–ECM communication and prevent the invasion of aggressive cancer cells [33,147,148,156]. On the other hand, extensive binding to extracellular targets was likely to cause problems observed in the clinical trials of **12**, such as the binding to skin collagen that result in painful blisters [147,148]. In these trials, **12** was administered by conventional intravenous injections. It is possible that administration of **12** by ITI could result in the predominant binding to the ECM that surrounds the tumor and to slow the spread of metastases, but this is yet to be established experimentally. More hydrophobic members of the Ru(III) series that have already undergone extensive preclinical development, such as **13a**,**b**, also have a potential for ITI, given that a suitable drug delivery formulation is used (see Section 7) [167]. The administration of such drugs directly into the tumor would result in rapid uptake by cancer cells and in cell death, while the formation of Ru-containing cell debris could lead to Ru–ECM binding and antimetastatic activity (Figure 6) [33,147,148,156].

The ability of certain metal complexes to promote the expression of damage-associated molecular patterns (DAMPs, Figure 6) on the surface of dying cancer cells, which leads to engagement of immune cells to the tumor (immunogenic activity), is crucial for the future of metal-based anticancer drugs [168,169]. Immunogenic properties have been demonstrated for many established anticancer drugs, including oxaliplatin, while cisplatin is generally considered to be non-immunogenic [168,169]. At least one Ru(III) compound (**13b**) has demonstrated the ability to induce immunogenic cancer cell death in vitro [151]. Such activity can provide an important additional benefit for the use of Ru(III) complexes in ITI [2,4,170]. Additional potential beneficial effects of the decomposition products of Ru(III) complexes used in ITI (Figure 6) include antimicrobial activity [165] and the disruption of the formation of amyloid aggregates, which are postulated to contribute to Alzheimer’s disease [154,155,166].

## 7. Drug Formulations for ITI

**Figure 7:**
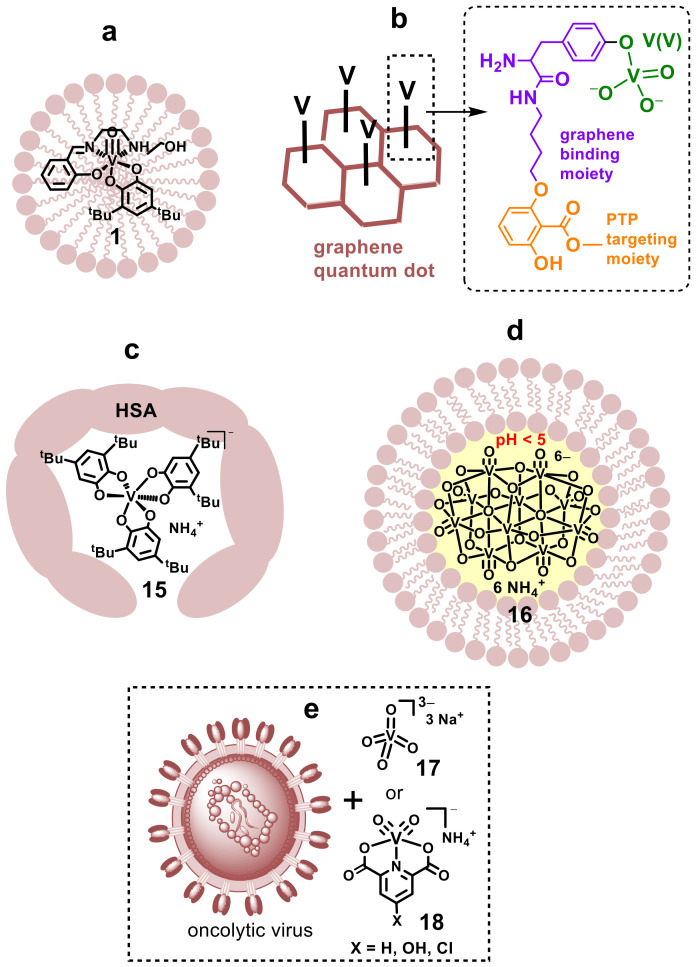
Potential pharmaceutical formulations for intratumoral injections of V(V) complexes (**1**, **15**–**18**): (**a**) hydrophobic micelles [174]; (**b**) protein tyrosine phosphatase (PTP)-targeting graphene quantum dots [175]; (**c**) adducts with human serum albumin (HSA) [177]; (**d**) pH-controlled liposomes [182]; and (**e**) co-administration with oncolytic viruses [187,188]. *^t^*Bu is *tert*-butyl.

Producing stable, injectable formulations of poorly water soluble and/or water-sensitive metal-based drugs is a significant challenge [49,167]. Many of the proposed ITI formulations of cytotoxic drugs, including Pt(II) complexes, involve polymeric matrices that are designed for the slow release of the drug [25,171,172,173], but these are less applicable to unstable metal complexes that have to be delivered rapidly. Some of the possible solutions that can be applied to unstable and reactive V(V) complexes, as well as to other metal complexes, include micellar systems (Figure 7a), graphene quantum dots (Figure 7b), human serum albumin (HSA) adducts (Figure 7c), liposomal systems (Figure 7d) and oncolytic virus–metal complex suspensions (Figure 7e).

A simple approach that is compatible with ITI involves the encapsulation of hydrophobic complexes, such as **1**, within micelles that are formed by a mixture of polyethylene glycol and fatty acids or triglycerides (Figure 7a) [174]. More recently, the binding of inorganic vanadate to small peptides that are incorporated into cell-permeable graphene quantum dots has been used for the precise delivery of V(V) to its cellular targets, such as a labile protein tyrosine phosphatase 1B (PTP1B) inhibitor, which was stabilized by the graphene framework (Figure 7b) [64,65]. This delivery system led to pronounced antidiabetic activity in mice [175]. Such technology also enabled the targeting of the compound using protein tyrosine phosphatases (protein tyrosine phosphatase 1B and T-cell protein phosphatase) [175]. Since applications of graphene quantum dots for selective anticancer therapy are under active development [176], a similar approach could potentially be designed for the delivery of unstable anticancer metal complexes to tumors via ITI techniques.

Another way to increase the aqueous solubility and stability of hydrophobic metal complexes, such as the V(V) tris-3,5-di-*tert*-butylcatecholato complex **15**, is to enclose them in hydrophobic pockets of human serum albumin (HSA, Figure 7c) [177]. The use of HSA as a carrier of anticancer drugs is expected to assist their retention in tumors [178] and a formulation using a HSA adduct of a Pt(IV) complex has entered human clinical trials [179]. In a related approach, the binding of inorganic V(V) and V(IV) salts to HSA through a covalently attached chelating ligand (EDTA) led to their efficient cellular uptake through caveolae-mediated endocytosis and high antiproliferative activity in cultured cancer cells [180]. This approach can potentially be used for the development of metal-ligand-HSA conjugates with an optimized lifetime for ITI applications [57].

Liposomal formulations of immunomodulating drugs are widely applied for use with ITI [181]. Water-soluble complexes, such as ammonium decavanadate **16**, or other polyoxometalates, can be encapsulated within unilamellar liposomes (Figure 7d) [182]. The pH value within the liposomes can be regulated to increase the stability of such complexes (Figure 7d) [183]. This approach may open the way for the wider use of unique biological activities of polyoxometalates that are different from those of mononuclear metal complexes [182,184,185]. Liposomal formulations have also been developed to enhance the stability of hydrophobic V(V) complexes in biological media [92]. 

A novel and highly promising way to harness the effect of V complexes on cellular signal transduction [66,70,72,186] is their use in enhancing the effects of oncolytic viruses [187]. Co-administration of a virus with inorganic vanadate (**17** in Figure 7e) or selected V complexes enhanced their uptake and cytotoxicity in cultured cancer cells and reduced tumor sizes in mice [187]. Viral infection and cytotoxicity in cancer cells was further enhanced by using more lipophilic V(V) complexes with dipicolinate ligands (**18** in Figure 7d) [188], although such complexes are known to be short-lived in aqueous solutions [189]. These findings are of immediate interest for the use in ITI of oncolytic viruses, which is the only ITI application currently approved for clinical use [8].

**Figure 8:**
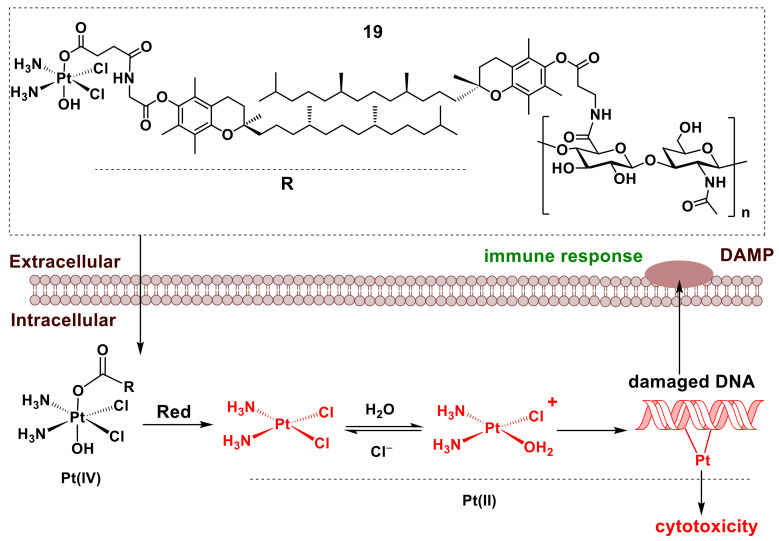
Structure of a Pt(IV)-tocopherol-hyalouronan nanocarrier **19** [190] and its proposed mechanism of action in ITI (based on a general mechanism of anticancer activity of Pt(IV) complexes) [191]. Proposed cytotoxic species are shown in red, and the beneficial immunogenic activity [168,190] is shown in green. Designations: Red are cellular reductants (e.g., ascorbate or glutathione), and DAMP are damage associated molecular patterns.

Injections of well-known cytotoxic Pt(II) complexes [15] directly into the tumor have been extensively trialed (Table 1) [7] to reduce their systemic toxicity compared with standard intravenous injections. However, significant side effects can still occur due to the partial escape of Pt(II) species into the blood stream [18,26,190]. To overcome this problem, a Pt(IV)-based nanocarrier formulation for ITI was developed recently (**19** in Figure 8) [190]. The formulation consists of a Pt(IV)-tocopherol derivative that is bound non-covalently (through hydrophobic interactions) to a hyaluronan-tocopherol adduct (Figure 8) [190]. The resulting nanoparticles are efficiently taken up by cancer cells and reduced by cellular reductants, such as glutathione and ascorbate, to form reactive Pt(II) species (marked with red color in Figure 8) [190,191]. These species enter the cell nucleus and form irreparable Pt(II)-DNA adducts, leading to cell death [15,191]. Importantly, the expressions of DAMPs on the surface of dying cancer cells [168,169] leads to the engagement of immune cells to the tumor and enhances the anticancer activity of **19** in an immunocompetent mouse model [190]. This immunogenic activity provides an additional benefit of using **19** for ITI (shown in green color in Figure 8) [31,181,190]. This example further demonstrates the potential of nanocarrier formulations in enhancing the activity and selectivity of metal complexes for ITI applications.

## 8. Conclusions and Future Potential Applications

Metal-based anticancer drugs [113,149,192] often have low stability in biological media [36,37,38,39,40,41,42], and this is one of the main obstacles to their wider use in clinical practice. A recent suggestion [57] was to take advantage of this instability and consequently reactivity and use these compounds in ITI applications (Figure 1). This novel concept was based on the results of in vitro stability studies and cell culture assays using a mixed-ligand V(V) complex showing significantly enhanced activity over cisplatin, **1** (Figure 2a) [57]. This literature survey highlights other metal-based anticancer drugs that could potentially be suitable candidates for ITI injections. Particularly, it focuses on considering anticancer Ti(IV), Ga(III) and Ru(III) complexes that were previously tested in human clinical trials but failed, which was attributed, at least in part, to the low stability when injected into the bloodstream [113]. 

The question posed for the compounds identified in this review (or related systems) is whether they would have the desired reactivity and sufficient stability to be useful for ITI applications. This approach has been used successfully in clinical trials with established Pt(II) drugs, mostly cisplatin (Table 1) [7] and in pre-clinical studies using a Pt(IV) prodrug [190]. In cell culture models [57,87], V(V) complexes with hydrophobic organic ligands were far superior to cisplatin in causing cancer cell death, particularly in short-term treatments that are relevant to ITI. The use of these biologically active but relatively unstable V(V) complexes can be further enhanced by the development of suitable drug formulations that stabilize the compounds further (Section 7). This is particularly relevant for their use in ITI and CED for the treatment of malignant gliomas [29,30,193]. Based on the low acute toxicity of **1** in healthy mice [83], the next logical step is the use of stabilized formulations of **1** and other hydrophobic metal complexes for intratumoral injections in mouse models of human cancers. These would use similar procedures to those used in previous studies with Pt(II) and Pt(IV) complexes [26,190]. The use of immunocompetent animals is particularly important for the assessment of immunogenic activity of **1** and other metal complexes [31,190]. 

Successful ITI has a cellular uptake of metal drugs that is faster than the extracellular complex decomposition. Since the proposed ITI approach is dependent on the kinetic competition between cellular uptake and extracellular decomposition, and this is characteristic for transition metal complexes, these complexes are ideal for such ITI applications (Figure 2, Figure 3, Figure 4, Figure 5 and Figure 6) [33,36]. In addition, Pt(IV) and Co(III) prodrugs that are activated by the reduction in the hypoxic environment of solid tumors (Figure 8) [49,50,51,53,56,190] can benefit from ITI by avoiding reduction in red blood cells before reaching the tumor target [54,55,56]. Generally, any cytotoxic metal complex can be considered for the use in ITI if it decomposes in an extracellular medium at a comparable rate with its cellular uptake and the decomposition products show lower toxicity compared with the initial complex [57]. The latter consideration is crucial to exclude the possibility that the cytotoxicity of the metal complex is due to the release of stable and biologically active ligands either inside or outside of the cell, such as **2** in Figure 2b or **11** in Figure 5 [86,90,91]. Under the conditions of ITI, the release of such ligands into the blood stream (Figure 1) is likely to lead to high systemic toxicity. Therefore, metal complexes of the ligands that have limited lifetimes in neutral aqueous solutions, such as Schiff bases (Figure 2a and Figure 3), can be particularly suitable for the use in ITI. More research is urgently needed to follow early kinetic studies [95,96] on the decomposition of such ligands and their complexes under biologically relevant conditions as well as methods that will stabilize these systems and facilitate the administration of these complexes. 

An important novel consideration in the use of metal complexes as anticancer drugs for ITI is the potential beneficial activity of their decomposition products (shown in green color in Figure 1, Figure 2, Figure 3, Figure 4, Figure 5, Figure 6 and Figure 8), which is unlikely to occur for non-metal-based drugs. Some of the most promising examples include the following: (i) immunogenic activities of some Pt(II), Pt(IV) and Ru(III) complexes [151,168,190]; antidiabetic, tissue regeneration and neurostimulatory activities of V(V/IV) complexes [63,78,79,81,82]; antimicrobial activities of Ga(III) [136,137,138,140,141,142,143], V(V/IV) [194,195] and Ru(III) complexes [165]; and antimetastatic and possibly neuroprotective activity of Ru(III) complexes [147,154,155,156,166]. The multiple modes of biological activity of many metal ions, dependent on their concentration and speciation in biological compartments [33,35,36,42,196] highlight the unique potential for metal complexes in medicinal applications, which is far from being fully realized at this time [49,52,113].

## Figures and Tables

**Table 1:** Current and recent clinical trials of ITI and related techniques using Pt-based drugs [7].

Identifier No	Treatment *	Drug	Disease	Phase	No. of Participants	Institution	Dates **
NCT04311762	ITI	cisplatin	stage IV lung cancer	I	9	University of Vermont, Burlington, VT, USA	February 2020–March 2022
NCT04809103	ITI	cisplatin	non-small cell lung cancer	I	10	University of Vermont, Burlington, VT, USA	March 2021–September 2023
NCT05200650	ITI	cisplatin- loaded gel	head and neck cancer	I	20	Hadassah Medical Center, Jerusalem, Israel	March 2022–November 2022
NCT04781725	ITI	new cisplatin formulation (INT230-6)	breast cancer	II	90	The Ottawa Hospital Research Institute and Cancer Center, Ontario, Canada	March 2021–March 2023
NCT01644955	CED	carboplatin	recurrent high-grade gliomas	I	10	Ohio State University Medical center, Columbus, OH, USA	June 2012–December 2017
NCT03294252	PIPAC	oxaliplatin and L-folinic acid	nonresectable peritoneal metastases of digestive cancers	II	50	Centre Hospitalier Lyon Sud, Pierre-Bénite, France	May 2017–June 2021
NCT04541108	ITI	carboplatin (various formulations)	development of master protocol for intratumoral microdosing	0	36	Presage Biosciences (various locations in USA)	July 2021–December 2031

* ITI = intratumoral injection; CED = convection enhanced delivery; PIPAC = pressurized intraperitoneal aerosolized chemotherapy. ** Start and end dates.

## Data Availability

Not applicable.
